# Short- and Mid-Term Survival of Geriatric Patients with Septic Arthritis of the Knee and the Impact of Risk Factors on Survival

**DOI:** 10.3390/jcm11030755

**Published:** 2022-01-30

**Authors:** Nina Pauline Haag, Markus Geßlein, Michael Millrose, Renate Ziegler, Maximilian Willauschus, Jörg Steinmann, Hermann Josef Bail, Johannes Rüther

**Affiliations:** 1 Department of Orthopedics and Traumatology, Paracelsus Medical University, 90471 Nuremberg, Germany; nina.p.haag@gmail.com (N.P.H.); markus.gesslein@klinikum-nuernberg.de (M.G.); m.millrose@icloud.com (M.M.); maximilian.willauschus@klinikum-nuernberg.de (M.W.); hermann-josef.bail@klinikum-nuernberg.de (H.J.B.); 2 Department of Trauma Surgery and Sports Medicine, Garmisch-Partenkirchen Medical Centre, 82467 Garmisch-Partenkirchen, Germany; 3 Institute of Clinical Hygiene, Medical Microbiology and Infectiology, Paracelsus Medical University, 90471 Nuremberg, Germany; renate.ziegler@klinikum-nuernberg.de (R.Z.); joerg.steinmann@klinikum-nuernberg.de (J.S.)

**Keywords:** septic arthritis, knee, geriatric patients, infection, risk factors

## Abstract

Septic arthritis is common in older adults and can be related to joint surgery or hematogenous distribution. To date, the risk factors affecting survival are unknown. This study aimed to evaluate the effects of existing implants, positive synovial microbiological culture results, and the American Society of Anesthesiology Physical Status (ASA) classification on the short- and mid-term survival of older patients with primary septic gonarthritis. This retrospective study included 133 older adults >60 years who underwent surgery for primary septic gonarthritis. Data were collected from medical records and public obituaries. Kaplan–Meier survival curves were used to estimate the probability of survival, as well as log-rank tests to measure and compare survival rates over one- and five-year periods. The mean age was 74.9 years (SD ± 9.2), and the 5-year follow-up rate was 74.3% (the mean follow-up was 3000.5 days; SD ± 1771.6). Mean survival was significantly different in patients with implants and without implants (*p* = 0.015), and between ASA II, ASA III, and ASA IV (*p* < 0.001). There was no significant difference in the survival of patients with or without a positive synovial microbiological culture (*p* = 0.08). Older adults with septic monoarthritis and pre-existing medical implants showed impaired survival. The ASA classification prior to surgery for primary septic monoarthritis can be helpful in identifying patients with poorer mid-term outcomes.

## 1. Introduction

Septic arthritis is predominantly found in older adults and children and involves large joints such as the knees, hips, and shoulders [1]. The causes of septic arthritis appear to be mainly exogenous, such as injury, iatrogenic injections, and invasive procedures [1,2,3]. The endogenous inoculation of bacteria from pre-existing bacteremia is also described as a cause of septic arthritis [1,2,3].

The role of implanted joint prostheses as a reservoir for bacterial infections remains unclear [4,5]. However, septic arthritis in joints with arthroplasties is associated with increased morbidity and poor functional outcomes [6].

Increasing age in patients with septic arthritis is also considered a risk factor for developing complications, recurrences, an increased duration of immobility, and extended hospital stays in older patients diagnosed with septic arthritis [6,7]. It is believed that an inadequate immune response may make older adults more susceptible to serious illness and death from sepsis [8].

Other risk factors that predispose the development of septic arthritis include previous surgery, osteoarthritis, diabetes mellitus, local skin infections, and intra-articular corticosteroid injections [2,7,9].

Organisms that are risk factors are mostly Gram-positive bacteria deriving from the human skin such as *Staphylococcus aureus* [2,6,10,11]. Since it is regularly seen that patients with biofilm formation show infections at distant locations, bacteria seem to have the ability to detach from the extracellular network, which suggests genetic variability within super- and non-biofilm formers [12]. This is crucial as implants provide surfaces for biofilm formation and, therefore, bacterial dissemination [13,14].

Surgical intervention as a treatment strategy is very beneficial and more effective than conservative therapy for patients diagnosed with septic arthritis [2,6,7,15]. Arthroscopic as well as open-joint debridement was found to improve recovery in addition to an improved long-term postoperative range of motion, a reduced requirement for blood transfusions, fewer complications, higher home discharge rates, and fewer revisions [2,16].

The American Society of Anesthesiology Physical Status (ASA) classification consists of six different stages ranging from a healthy patient (Stage I) to a brain-dead patient (Stage VI). The classification method is well documented and has proven to be a reliable representative predictor of a patient’s outcome, complications, and healing process [17,18]. The purpose of the classification is to assess and communicate a patient’s pre-anesthesia medical comorbidities. The ASA correlates significantly with operating times, length of hospital stays, postoperative infection rates, and morbidity and mortality rates [19].

The purpose of this study was to analyze the overall survival of older adults with acute primary septic monoarthritis of the knee joint following surgical treatment and to evaluate possible risk factors that affect patient survival. It was hypothesized that medical implants in other parts of the body or positive synovial microbiological cultures would impact the survival of older adults with septic arthritis. Further, it was hypothesized that an ASA score greater than III at the time of surgery may be associated with reduced survival in older adults with septic arthritis.

## 2. Materials and Methods

This retrospective cohort study was designed to evaluate the survival rates of older patients with primary septic arthritis, in conjunction with the role of medical implants in various parts of the body, synovial microbiological cultures, and the ASA PS classification.

Patients were identified via clinical records from specialized medical care hospitals for the period of 1 January 2007 to 31 October 2020. A total number of 238 patients were admitted to the emergency department for septic arthritis of the knee. There were 179 patients included in the study, and a complete follow-up was completed for 133 of those patients (74.3%).

The inclusion criteria were: an age above 60 years, primary septic monoarthritis of the knee joint, and meeting at least one of the Newman criteria for septic arthritis [11].

The exclusion criteria were: periprosthetic joint infection, incomplete medical records, unavailable survival data, recent surgery (<6 months), open skin wounds at the knee or general wound treatment, and a diagnosis of acute gout, rheumatoid arthritis, or crystal arthropathies.

Treatment of the patients started immediately after a diagnosis of septic arthritis as an emergency procedure and ended when patients either died or presented with a normal CRP (normal reference < 0.5 mg/dL), normal leukocyte count (normal reference range 4.5–10 × 10^3^ cells/mcL), and no fever for 48 h (<38.5 degrees Celsius, auricular).

Diagnostic work-up included: hospital admission, a clinical examination, blood work, a plain joint X-ray, sterile joint aspiration in the emergency department for microbiological evaluation, joint lavage with microbiological sampling during surgery, empiric antibiotic treatment, and directed antibiotic therapy after microbial testing results.

Recorded factors included: age in years, sex, the affected joints, the existence and site of implants (cardiovascular, urogenital, respiratory, gastrointestinal, or orthopedic), the date of hospital admission, clinical examination methods, blood parameters (including leukocyte count, CRP), time until follow-up in days, the date of death, the ASA PS classification scores at the time of surgery [17,18], microbiological blood culture analyses, and the number of joint surgeries during the hospital stay.

Joint aspiration was performed by an orthopedic surgeon according to a standardized aseptic technique and was then sent for aerobic/anaerobic growth. Synovial fluid was inoculated on aerobic chocolate, sheep blood agar, and anaerobic sheep blood agar plates then incubated for 7 days aerobically at 37 °C with 5% CO_2_ and anaerobically at 37 °C. The remaining fluid was inoculated in thioglycolate broth for sample enrichment and then incubated for 14 days.

The identification of bacteria was carried out using matrix-assisted laser desorption/ionization—time of flight mass spectrometry (MALDI-TOF MS). Antibiotic susceptibility testing was performed according to the European Committee on Antimicrobial Susceptibility Testing (EUCAST).

Follow-up and survival data were collected from subsequent medical records, publicly announced obituaries in local newspapers, and online archives.

The study was approved by a university research ethics board. All data were collected and analyzed anonymously.

Statistical analysis was performed using IBM SPSS Statistics version 26. The p-value was considered significant with values of *p* ≤ 0.05.

Kaplan–Meier survival curves were used to estimate the probability of a patient’s survival over a five-year timeline, separately for each of the following groups: older adults with septic arthritis with and without medical implants, those with positive and non-positive synovial sample cultures, and those grouped according to their presurgical ASA PS classification scores (ASA II, ASA III, or ASA IV).

A log-rank test was used to compare the probabilities of survival from the Kaplan–Meier survival curves for each of the groups: implants, synovial sample cultures, and presurgical ASA PS classifications. First-year mortality rates were determined using life tables from the Kaplan–Meier survival curves. Univariate analysis was performed and is reported as the mean, median, standard deviation, and confidence interval, unless otherwise stated.

## 3. Results

Detailed patient characteristics divided by sex are summarized in Table 1.

The mean follow-up for all patients was 3000.5 days (SD ± 1771.6; range 10–5095 days) or 8.22 years, with a follow-up rate of 74.3%. A total of 102 (76.7%) patients survived the first year. A total of 63 (47.4%) patients survived the first five years after their initial diagnosis of septic arthritis.

### 3.1. Survival Data of Patients—Medical Implants in Other Areas of the Body

**Figure 1:**
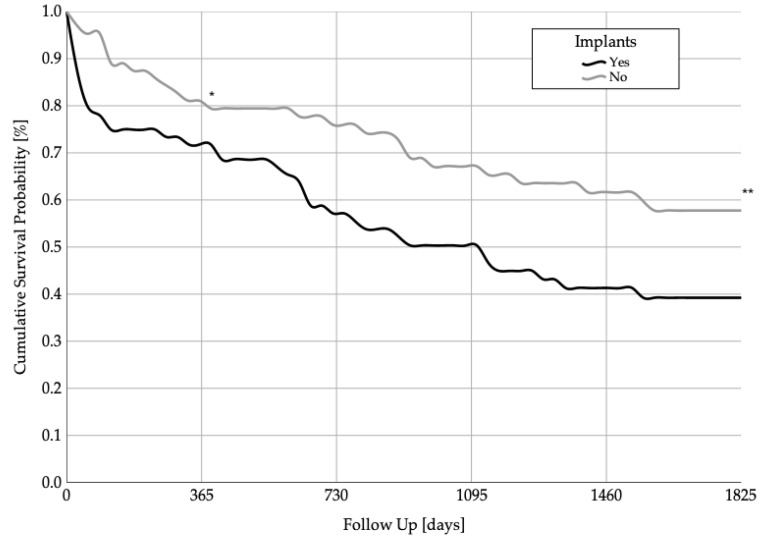
Cumulative 1-year * (*p* = 0.031) and 5-year ** (*p* = 0.015) survival probabilities of patients with pre-existing medical implants and patients without pre-existing medical implants.

Patients with medical implants had a mean survival rate of 2112.5 ± 261.5 days (95% CI 1599.9–2625.0), while the mean survival rate in patients without implants was 3255.9 ± 291.7 days (95% CI 2684.3–3827.7). The 1-year * (*p* = 0.031) and 5-year ** survival rates (*p* = 0.015) were statistically significantly different between the two groups. The Kaplan–Meier survival analysis is shown in Figure 1. At the 1-year follow-up, 82% of patients without implants and 71% of patients with implants were still alive. At the 5-year follow-up, 40% of patients with implants and 59% of patients without implants were still alive.

### 3.2. Survival Data of Patients—Synovial Microbiological Culture

Twenty-two different causative organisms were identified; fourteen (63.6%) were classified as Gram positive and eight (36.4%) were classified as Gram negative, of which *Staphylococcus aureus* was the most common (32.3%). Further detailed information can be found in Table 2.

**Figure 2:**
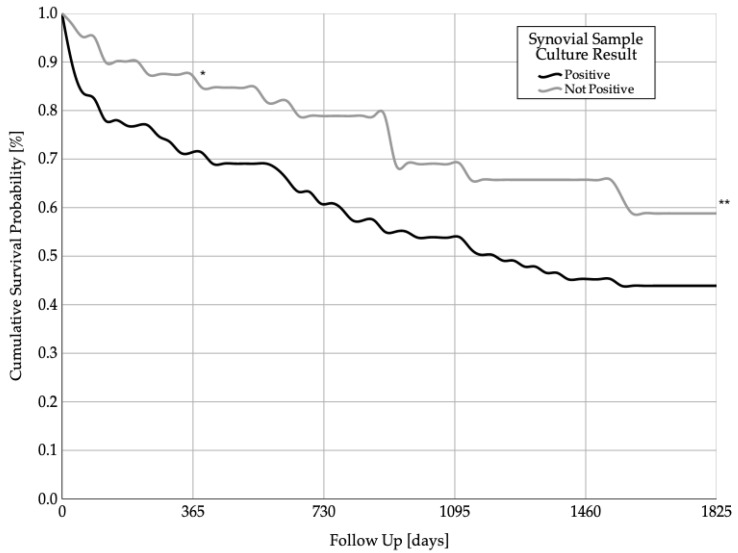
Cumulative 1-year * (*p* = 0.24) and 5-year ** (*p* = 0.08) survival probabilities of patients with positive synovial microbiological culture results and patients without a positive result.

The mean survival of patients with a positive synovial microbiological culture was 2541.2 ± 250.0 days (95% confidence interval (CI) 2051.4–3031.1), while the mean survival in patients without a positive synovial microbiological culture was 2759.5 ± 300.6 days (95% CI 2170.3–3348.6). The 1-year * (*p* = 0.24) and 5-year ** (*p* = 0.08) survival rates were not statistically significantly different between the two groups. The Kaplan–Meier survival analysis is shown in Figure 2.

At the 1-year follow-up, 88% of patients without a positive synovial sample culture and 72% of patients with a positive synovial sample culture were still alive. At the 5-year follow-up, 43% of patients with a positive synovial sample culture result and 59% of patients without a positive synovial sample culture result were still alive.

### 3.3. Survival Data of Patients—ASA Physical Status Classification

**Figure 3:**
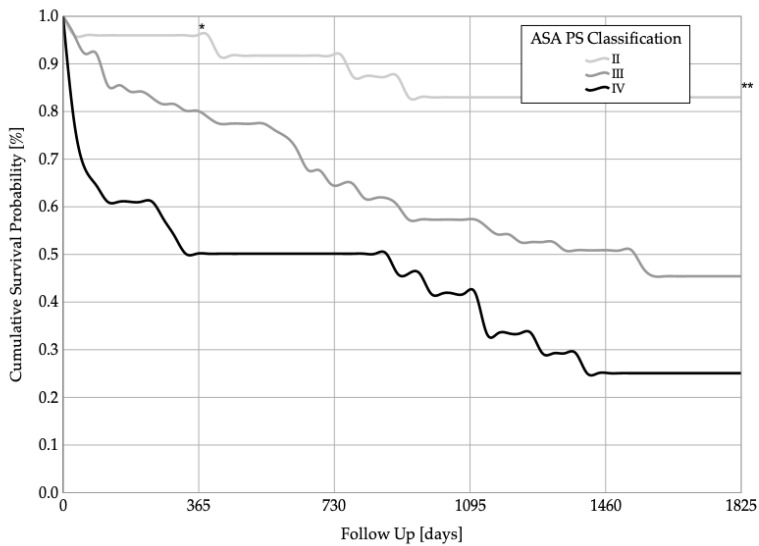
Cumulative 1-year * (*p* < 0.001) and 5-year ** (*p* < 0.001) survival probabilities of patients categorized as ASA PS classifications II–IV.

The mean survival rate in patients categorized as ASA II was 4324.1 ± 352.4 days (95% CI 3633.4–5014.7), while the mean survival rate in patients categorized as ASA III was 2438.4 ± 244.4 days (95% CI 1959.5–2917.4), and the mean survival rate in patients categorized as ASA IV was 1533.8 ± 375.9 days (95% CI 797.0–2270.5). The 1-year * (*p* < 0.001) and 5-year ** survival rates (*p* < 0.001) were statistically significantly different between the groups. The Kaplan–Meier survival analysis is shown in Figure 3. At the 1-year follow-up, 97% of ASA II patients, 80% of ASA III patients, and 50% of ASA IV patients were still alive. At the 5-year follow-up, 83% of ASA II patients, 45% of ASA III patients, and 25% of ASA IV patients were still alive.

## 4. Discussion

The main findings of this study are that survival was found to be significantly impaired in older adults with septic gonarthritis and pre-existing medical implants, compared to older adults with septic gonarthritis who did not have implants. Moreover, older adults categorized as ASA II–ASA IV showed significantly decreased survival rates at the higher ASA PS classifications. No significant differences in survival were found in older adults with and without a positive synovial microbiological culture.

There are scarce data on populations of older patients with septic arthritis, and many studies lack a mid-term or long-term follow-up period. The results from this intensive case series over a mean follow-up period of more than 8 years suggest that medical implants anywhere in the body may contribute to an alteration in the mid-term survival rates of older adults. Previous studies showed a similar trend [6,7,15]. Short-term follow-ups indicate the same trend as this study. This study did not fully evaluate the reasons behind this trend in comprehensive detail. Patients needing an implant are more likely to suffer from other more severe diseases. Therefore, their overall health may be compromised to a greater extent. This may lead to a higher susceptibility to infections as well as higher mortality rates. However, secondary site infections due to biofilm formation around an implant and associated bacterial dissemination are factors to be considered. Otto et al. [13] described both *Staphylococcus aureus* and *Staphylococcus epidermidis* as showing the genetic characteristics and phenotypical expressions for biofilm formation. Further, a stronger inflammatory response induced by biofilm-released cells versus planktonic cells has been described [20]. This may explain why the primary bacterial invasion and biofilm formation did not induce a septic reaction, but the secondary site infection did. Takahashi et al. [21] described *Staphylococcus aureus* as the causative organism and an important prognostic factor for the outcome of septic arthritis. As *Staphylococcus aureus* was found to be most common in this study, this might further clarify the relationship between pre-existing medical implants and survival rates.

Patients within the ASA IV classification presented with the highest mortality. These results align with previous studies, suggesting the comparability of the data, and the significant ability to predict postoperative outcomes from the preoperative ASA PS classifications assigned to patients [19]. Thus, the existence of severe systematic diseases that are a constant threat to life is present when classified as ASA IV. This suggests a higher risk of death as a final outcome [18]. Therefore, the results of this study indicate that older patients with implants and an ASA classification greater than ASA III might require a higher level of intensive care therapy than previously presumed.

Comparison with other studies is challenging as mid-term to long-term follow-ups of operative studies are rare, and mortality follow-ups are uncommon. However, 1-year mortality follow-ups are found more frequently. In this study, the 1-year mortality rate of patients with septic arthritis and implants was 28.2%. In patients with septic arthritis and a positive culture, the 1-year mortality was 28.7%. Other orthopedic conditions show mortality rates lower than Myers et al.’s model suggests in their study of older adults with distal femur fractures and a 1-year mortality rate of 13.4% [11,22].

### Limitations

Limitations regarding the broader applicability, scientific accuracy, and interpretation of results such as *p*-values and confidence intervals arise from the single-center and retrospective study method [23]. However, this method was chosen as septic arthritis often appears as an emergency procedure. This can impede prospective designs and any required preparations.

Age is a significant risk factor for osteoarthritis, especially in the knees. This study did not evaluate the presence of osteoarthritis in other joints [6,7]. Patients diagnosed with other instances of arthritis such as gout or pseudogout were excluded from the study. However, the presence of crystals in the synovial fluid does not rule out possible infection. Therefore, we may have excluded adequate patients who were potentially misdiagnosed with a primary diagnosis of crystal arthropathy and secondary septic arthritis.

Selection bias can be assumed as the 5-year follow-up of 74.3% may have influenced survival rates. It remains unknown whether patients lost to follow-up survived or not. Further, in an older population with different comorbidities, which may not have been addressed in detail, it can be assumed that there are confounding factors.

Actual death or survival was the only outcome included in the data. Underlying conditions, medications, and functional outcomes (except for diabetes mellitus) were not incorporated into the study. Individual direct causes of death also remain unknown, limiting the follow-up to a great extent.

Additionally, performance bias must be considered as surgical procedures were performed by multiple surgeons.

The strength of this study is that risk factors affecting mid-term survival in patients with septic arthritis were observed in conjunction with one another rather than individually, and follow-ups could be achieved over a long period with the majority of patients.

## 5. Conclusions

Older adults diagnosed with septic monoarthritis and who had pre-existing medical implants showed impaired survival rates. The ASA classification prior to surgery can be helpful in identifying patients with lower mid-term outcomes and can improve survival, therefore providing an alert that additional services are needed to improve survival ratios.

## Figures and Tables

**Table 1:** Characteristics of the patients by sex.

	Sex		Total
	Female	Male	
Number of patients, *n* (%)	48 (36.1)	85 (63.9)	133 (100)
Mean age in years, SD (range)	75.9 ± 10.0 (60–96)	74.3 ± 8.7 (60–92)	74.9 ± 9.2 (60–96)
Positive synovial microbiological culture, *n* (%)	33 (24.8)	58 (43.6)	91 (68.4)
Diabetes mellitus, *n* (%)	14 (10.5)	30 (22.6)	44 (33.1)
Implants in other areas, *n* (%)			
Arthroplasty of Other Joints	15 (21.4)	22 (31.4)	37 (52.8)
Intravascular Device	8 (11.4)	12 (17.1)	20 (28.6)
Fracture Fixation Device	2 (2.9)	4 (5.7)	6 (8.6)
Urogenital	1 (1.4)	4 (5.7)	5 (7.1)
Respiratory	0 (0)	1 (1.4)	1 (1.4)
Gastrointestinal	0 (0)	1 (1.4)	1 (1.4)
Total	26 (37.1)	44 (62.9)	70 (100)
ASA PS classification, *n* (%)			
ASA I	0 (0)	0 (0)	0 (0)
ASA II	8 (6.0)	17 (12.8)	25 (18.8)
ASA III	32 (24.1)	44 (33.1)	76 (57.1)
ASA IV	8 (6.0)	24 (18.0)	32 (24.1)
Number of surgeries, *n* (%)			
1	24 (50.0)	48 (56.5)	72 (54.1)
2	15 (31.3)	29 (34.1)	44 (33.1)
3	6 (12.5)	5 (5.9)	11 (8.3)
4	2 (4.2)	3 (3.5)	5 (5.9)
5	1 (2.1)	0 (0)	1 (0.8)

*n* = number of patients; % = percentage; ASA PS classification = the American Society of Anesthesiology Physical Status classification; SD = standard deviation.

**Table 2:** Distribution of pathogens.

Pathogen	Number, *n* (%)
**Gram positive**	
*Corynebacterium aurimucosum*	1 (0.8)
*Propionibacterium acnes*	1 (0.8)
*Total Staphylococcus family*	58 (43.6)
*Staphylococcus aureus*	43 (32.3)
*MRSA*	5 (3.8)
*Streptococcus agalactiae*	4 (3.0)
*Streptococcus dysgalactiae*	4 (3.0)
*Streptococcus equi*	1 (0.8)
*Streptococcus pneumoniae*	1 (0.8)
*Streptococcus pyogenes*	2 (1.5)
*Viridans Streptococci*	1 (0.8)
*Total gram positive*	73 (54.9)
**Gram negative**	
*Borrelia burgdorferi*	2 (1.5)
*Citrobacter koseri*	1 (0.8)
*Enterobacter cloacae*	2 (1.5)
*Enterococcus faecalis*	3 (2.3)
*Enterococcus faecium*	1 (0.8)
*Escherichia coli*	6 (4.5)
*Pseudomonas aeroginosa*	2 (1.5)
*Serratia marcescens*	1 (0.8)
*Total gram negative*	18 (13.5)
Negative synovial microbiological results	42 (31.6)
**Total**	**133 (100.0)**

MRSA = Methicillin-resistant Staphylococcus aureus.

## Data Availability

Data were obtained from a maximal care unit for surgery and traumatology at Klinikum Nuremberg Süd. Data can be provided anonymously upon request in a separate file. A public dataset was not used to obtain the presented data.
